# Nonpatient Stakeholder Perspectives on the Use of Gamification and Financial Incentives in Mobile Health for Medication Adherence: Modified Nominal Group Technique Study

**DOI:** 10.2196/101295

**Published:** 2026-10-29

**Authors:** Steven Tran, Lorraine Smith, Stephen Carter

**Affiliations:** 1 School of Pharmacy Faculty of Medicine and Health University of Sydney Camperdown Australia

**Keywords:** qualitative research, stakeholder perspectives, gamification, incentives, mobile app, mHealth, medication adherence, digital health, mobile health

## Abstract

**Background:**

Medication nonadherence remains a major global health challenge, contributing to preventable disease, hospitalizations, and health care costs. Mobile health (mHealth) apps incorporating gamification and financial incentives have shown potential to improve adherence; however, most research has focused on patient perspectives, with limited understanding of how nonpatient stakeholders perceive their feasibility, risks, and implementation. Understanding nonpatient stakeholder perspectives in relation to patient viewpoints is essential for informing future policy development and establishing practical, industry-supported safeguards that protect consumers while enabling innovation.

**Objective:**

This study aimed to explore nonpatient stakeholder perspectives on the use of gamification and financial incentives in mHealth apps for medication adherence and to inform consensus-based design and policy considerations.

**Methods:**

A study was conducted using a modified virtual nominal group technique (vNGT). Nonpatient stakeholders across health care, industry, and policy sectors in Australia were recruited through purposive and convenience sampling between February and August 2025. Data collection involved a survey capturing demographics and initial perspectives, followed by online focus groups for consensus discussions. Qualitative responses were analyzed using thematic analysis supported by AI-assisted coding. Consensus statements derived from themes were rated during the focus groups. Additional probing prompts were used in the focus groups to encourage further discussion where consensus was not immediately achieved. Results from the focus groups were analyzed using descriptive statistics to summarize consensus ratings and discussions.

**Results:**

A total of 20 participants were included in the study. Nine participants reported having a health care professional background employment exclusively, while 11 reported having employment background across multiple sectors or exclusively outside health care. Six key themes were identified: tailored gamification for adherence, financial incentives as a contested motivator, designing for diversity and inclusion, usability barriers to engagement, trust through data governance, and validated and sustainable innovation. These informed 24 consensus statements, of which 54% (13/24) achieved unanimous agreement. Stakeholders strongly endorsed personalization, simplicity, and transparent data practices, while expressing nuanced concerns regarding the ethical use, sustainability, and potential unintended consequences of financial incentives.

**Conclusions:**

Nonpatient stakeholders largely reinforce patient priorities while extending them with critical perspectives on implementation, governance, and sustainability. Gamification and financial incentives are viewed as potentially effective but require careful, ethically grounded design to balance engagement with long-term motivation and trust. These findings support the development of stakeholder-informed guidelines for responsible mHealth innovation and highlight the importance of integrating patient and system-level perspectives in digital health design. Future research should prioritize co-designed longitudinal studies using apps with gamification and a range of incentive offers with clear redemption processes to evaluate the long-term impact on medication adherence across diverse patient populations.

## Introduction

### Background

Medication nonadherence continues to pose a significant challenge to global health systems, contributing to preventable disease progression, increased hospitalizations, and substantial economic costs [1,2]. It is estimated that nearly half of all patients with chronic conditions fail to take their medications as prescribed, leading to avoidable complications and billions of dollars in health care expenditures annually [1,2]. This persistent issue undermines treatment efficacy and places a considerable burden on health care infrastructure. Addressing medication nonadherence requires scalable, patient-centered interventions that can support sustained behavioral change across diverse populations.

Mobile health (mHealth) apps have emerged as promising tools to address this challenge by delivering personalized, real-time interventions through smartphones and other digital platforms [3]. These technologies offer flexible and accessible means of engaging patients in their treatment regimens, often incorporating behavioral strategies such as reminders, educational content, and interactive features. Among these strategies, gamification and financial incentives have gained increasing attention for their potential to enhance user engagement, motivation, and adherence [4,5].

Gamification refers to the application of game-like elements such as points, badges, leaderboards, and challenges within nongame contexts, aiming to encourage behavior change through both intrinsic and extrinsic motivation [6]. In mHealth, these elements are designed to make adherence-related tasks more engaging, rewarding, and socially interactive. Financial incentives, by contrast, provide tangible rewards (eg, vouchers, discounts, or monetary compensation) for adherence behaviors, drawing on behavioral economic principles that emphasize the reinforcing power of immediate rewards in shaping long-term habits [7,8]. Both approaches have demonstrated promise in improving short-term adherence outcomes, particularly when integrated into digital platforms that allow for real-time tracking and feedback [5,9]. Prior studies have also identified gamification and financial incentives as tools to support various behavior change theories such as Ryan and Deci’s self-determination theory (SDT), which proposes that sustained motivation is fostered when interventions support individuals’ needs for autonomy, competence, and relatedness [5,10]. Accordingly, SDT provides a useful theoretical lens for interpreting how design features of mHealth interventions may influence engagement and medication adherence. Despite growing interest in these engagement strategies, their implementation within mHealth platforms remains a multifaceted challenge.

Although previous studies have explored stakeholder perspectives across a range of digital health technologies and implementation contexts [11-18], comparatively little attention has been given to how nonpatient stakeholders perceive the use of gamification and financial incentives in mHealth interventions for medication adherence. Qualitative studies exploring patient perspectives have shown that users generally value mHealth interventions that are personalized, easy to use, and supportive of their autonomy. The same studies have also highlighted concerns regarding privacy, inappropriate use of gamification, and the ethical use of financial incentives [11-15]. These studies have helped establish patient-centered design principles for mHealth interventions and demonstrate that the perceived value of gamification and financial incentives depends not only on whether they are used, but also on how they are designed and implemented [11].

In the field of mHealth development, a growing body of literature has explored the perspectives of nonpatient stakeholder groups involved in the design, implementation, and governance of digital health technologies. In this context, nonpatient stakeholders refer to health care professionals, industry providers, third-party health care payers, and policymakers. Nonpatient stakeholders provide unique insights into their perceptions of the opportunities, risks, and implementation challenges associated with gamification and financial incentives in medication adherence interventions. Nonpatient stakeholders share responsibility for clinical integration, governance, technical feasibility, reimbursement, and long-term sustainability [19,20].

Existing literature on various stakeholder groups suggests priorities that differ across professional roles. Health professionals cite the need for multidisciplinary collaboration as a cornerstone of effective digital health innovation [19]; yet, little is known about how health professionals would welcome collaboration around apps that use or rely on gamification and/or incentives. Health care professionals often acknowledge the potential of mHealth tools to support medication adherence but express concerns regarding clinical validation, usability, and data privacy [21]. Industry stakeholders, including app developers and digital health companies, emphasize the importance of user retention, regulatory compliance, and return on investment [22]. Meanwhile, insurers and policymakers shape the feasibility of financial incentives through reimbursement models and ethical frameworks [23]. Understanding where stakeholders’ priorities align or diverge may therefore inform the development of mHealth interventions that are not only acceptable to end users but are also feasible to implement within routine health care systems [24,25].

### Objectives

This study aims to explore the perspectives of nonpatient stakeholders on the integration of gamification and financial incentives in mHealth apps designed to support medication adherence. Specifically, the study seeks to (1) gather the perspectives of nonpatient stakeholders on the use of gamification and financial incentives in mHealth apps for medication adherence and (2) develop consensus statements on the use of gamification and financial incentives in mHealth apps, based on the perspectives of nonpatient stakeholders.

By engaging a diverse group of stakeholders, this study aims to generate actionable insights that inform the co-design of evidence-based, patient-centered, and stakeholder-aligned mHealth apps to support medication adherence.

## Methods

### Overview

This study used a modified virtual nominal group technique (vNGT) [26] with thematic analysis [27] and descriptive analysis of consensus ratings. This approach was modified by incorporating a survey-based idea generation phase prior to virtual focus groups to reduce the duration of synchronous sessions while preserving the core principles of idea generation, structured discussion, and consensus-building. This modification was considered appropriate given the anticipated time constraints of multidisciplinary professional stakeholders.

The study was conducted in 2 phases: a survey administered prior to focus groups (Phase 1), followed by online focus group sessions (Phase 2). Phase 1 supported idea generation and consolidation of the nonpatient stakeholder perspectives, while Phase 2 facilitated consensus-building through structured anonymous voting, feedback on results, and discussion. A high-level overview diagram of the study is available in Multimedia Appendix 1.

The COREQ (Consolidated Criteria for Reporting Qualitative Research) checklist was used to guide reporting [28]. A completed COREQ checklist applied to this study is available in Multimedia Appendix 2.

### Ethical Considerations

Ethical approval for this study was granted by the Human Research Ethics Committee (HREC) of The University of Sydney (2024/HE001141). All qualitative data were deidentified prior to analysis. No personally identifiable information or sensitive participant details were included in prompts. Data were processed in accordance with institutional ethical guidelines and relevant data governance standards.

### Recruitment

Participants were recruited using criterion-based purposive sampling supplemented by convenience sampling. Purposive sampling was used to identify individuals from predefined stakeholder groups within the Australian ecosystem, based on their expertise relevant to digital health. Recruitment sought to include representation from each stakeholder group. Participants were eligible for inclusion if they were aged 18 years or older and belonged to one of the predefined stakeholder groups: health care professionals, academics, government and nonprofit representatives, members of professional societies and associations, pharmaceutical and health insurance representatives, and experts in digital health law and ethics. Exclusion criteria included individuals not residing in Australia. Convenience sampling was subsequently used through professional networks and social media to broaden recruitment within these stakeholder groups. Two primary recruitment pathways were used:

1. Direct outreach via public and professional networks: potential participants were identified through publicly accessible sources and professional networks using the predefined stakeholder groups as selection criteria, based on their expertise and experiences relevant to digital health. Where contact details were publicly available, the first author (ST) initiated contact via email or phone. This initial communication included a brief introduction to the study, a secure REDCap (Vanderbilt University) survey link, and the participant information statement.
2. Social media and professional platforms: recruitment posts were disseminated through LinkedIn, targeting researchers and professionals within the predefined stakeholder groups. These posts contained a brief study description and the REDCap survey link. This convenience sampling approach broadened recruitment within the predefined stakeholder groups.

Interested individuals accessed the survey to read the context of the study, to undergo eligibility screening, to provide informed consent, and to complete the survey. As recruitment occurred over several months, the interval between survey completion and the commencement of the focus groups varied across participants. However, all surveys were completed and analyzed before any focus groups were conducted, allowing responses from the entire participant cohort to inform the subsequent focus group discussions. This process facilitated idea generation, in alignment with the principles of the vNGT as described by Lee et al [26].

### Phase 1: Survey Design

#### Overview

The instrument was developed specifically for this study and was informed by prior research on patient perspectives on the same topic [11]. Survey items (quantitative and qualitative questions) elicited participant characteristics as well as attitudes toward gamification and financial incentives in mHealth apps for medication adherence. For the demographic section, participants were able to select multiple stakeholder categories to reflect their current and past professional experiences, as well as their years of experience. Participants’ perspectives were explored using quantitative rating scales and open-ended questions. Patient-derived perspectives were incorporated as prompts to enable comparison between stakeholder and prior patient viewpoints. These prompts were designed to capture key domains identified in prior research, including personalization of gamification and incentives, motivational impact, usability and accessibility, ethical considerations, and data governance. The survey was not piloted, pretested with participants, nor was it formally validated. However, all authors reviewed the survey prior to administration to establish content and face validity. The survey is provided in Multimedia Appendix 3.

#### Quantitative: Rating Scale

Participants were asked to provide their level of agreement with 5 prompts derived from the key patient perspectives identified in our previous qualitative study [11]. These prompts represented key patient-identified domains, including (1) personalization of gamification and incentives, (2) concerns regarding financial incentives, (3) usability and complexity, (4) data privacy, and (5) customization. Participants rated each prompt using an 11-point agreement scale, where 0 indicated “strongly disagree” and 10 indicated “strongly agree.” No other words were used on the scale. For descriptive reporting, responses were categorized into 5 levels: 0-2 (“strongly disagree”), 3-4 (“disagree”), 5 (“neither agree nor disagree”), 6-7 (“agree”), and 8-10 (“strongly agree”).

#### Qualitative: Open-Ended Questions

Following the quantitative component, participants were asked to complete 11 open-ended questions designed to elicit further insights and perspectives regarding gamification and financial incentives in mHealth apps for medication adherence.

### Idea Consolidation

All authors (ST, SC, and LS) initially conducted familiarization exercises on the survey responses. The first 6 survey responses were independently coded by each author to calibrate the coding process. Coding differences were discussed collaboratively to achieve a consistent coding approach, including agreement on the level of granularity at which concepts should be coded. These discussions informed the development of a shared coding framework. The remaining data were coded by ST using NVivo (Release 1.7.2; Lumivero), with subsequent review by SC and LS to ensure consistency of coding. Individual codes representing meaningful concepts within participants’ responses were then grouped according to conceptual similarity into preliminary thematic nodes using Microsoft Excel (version 2506). These preliminary thematic nodes were iteratively reviewed, refined, and consolidated into broader themes through discussion among the authors before proceeding to the AI-assisted stages of analysis. Coding decisions, theme development, and subsequent refinements were documented throughout the analysis to provide an audit trail.

Survey responses to the qualitative questions were subsequently analyzed using generative AI to support thematic analysis and inform the development of consensus statements. This process followed a structured 6-phase approach which was adapted from Naeem et al [27]. The analysis combined manual coding with AI-assisted support using a university-provisioned instance of OpenAI’s GPT-5. The deployed model strictly prohibited the exfiltration of user prompts or operational data to third-party domains.

OpenAI’s GPT-5 use was integrated at subsequent stages of the analysis. Standardized AI prompts were developed and were applied consistently across the dataset. AI prompts used to guide the thematic analysis are provided in Multimedia Appendix 4.

To begin the use of AI assistance, the full dataset was input for AI-assisted familiarization and initial coding (steps 1 and 2). The AI reviewed the authors’ coding using the predefined prompts and identified 9 additional candidate codes not previously captured. Each candidate code was manually reviewed against the original dataset by the authors to determine its contextual relevance and distinctiveness before being incorporated into the final coding framework. AI then conducted theme identification (step 3), which was cross-validated against the authors’ manual node groupings. Any differences in interpretation were resolved through discussion and review of the original data. No substantive differences in theme structure or interpretation were identified. A second round of theme identification confirmed the same 6 themes.

Subsequent AI-assisted steps included theme review (step 4), definition and naming of themes (step 5), and preliminary report generation (step 6). The authors independently reviewed all AI-generated outputs after each step against the original dataset and relevant literature to validate thematic integrity and identify conceptual gaps, particularly around gamification and financial incentives. AI-generated outputs were treated as supplementary analytical inputs and were not incorporated without verification against the original dataset by the authors, thereby reducing the risk of hallucination or oversimplification. Final consensus statements and discussion prompts for the focus groups were developed from the agreed themes.

During the analytical process, themes were compared to the raw data iteratively to identify the emergence of new concepts relevant to the study objectives. Recruitment ceased when the 3 most recent survey responses did not contribute any additional concepts beyond those already identified, suggesting diminishing conceptual novelty and that sufficient breadth of perspectives had been captured to inform the subsequent focus group discussions [29].

### Reflexivity

The authors had expertise in pharmacy, medication adherence, digital health, and qualitative research. The authors recognized that their disciplinary backgrounds and prior experience with digital health interventions could influence interpretation of participant responses. The authors also acknowledged the potential impact of personal experiences with gamification and financial incentives in health and nonhealth domains on the facilitation and interpretation of the study. To promote reflexivity throughout analysis, potential assumptions and interpretations were discussed during regular meetings. Coding calibration was undertaken collaboratively, and coding decisions, theme development, and interpretation were reviewed by all authors to ensure that the findings remained grounded in participants’ responses rather than the perspective of a single researcher.

### Phase 2: Focus Group Sessions

Online focus group sessions, each comprising 4 to 6 participants, were conducted and facilitated by the first author (ST). The focus groups followed a facilitator guide incorporating a standardized discussion protocol, in which each proposed consensus statement was presented sequentially for discussion and voting. The facilitator guide is provided in Multimedia Appendix 5. The sessions were recorded and transcribed using Zoom’s (Zoom Communications, Inc) automated transcription function. The first author (ST) reviewed each transcript against the original recordings prior to analysis to verify their accuracy, consulting the recording where clarification was required. Participants were presented with the themes and proposed consensus statements derived from the survey and were invited to discuss and clarify the findings, including the analytical process used to develop the themes.

A structured anonymous voting method was used, using the polls functionality within Zoom, to vote (agree or disagree) on consensus statements derived from the thematic analysis of the survey responses. The same set of consensus statements was used across all focus group sessions. Following anonymous voting, the aggregated voting results were shared with participants. Where unanimous agreement was not immediately achieved, the facilitator directed probing prompts to the group as a whole (eg, “Could you tell me more about what has informed your perspective?”) to encourage further discussion and clarification of differing viewpoints. Participants were not required to disclose how they had voted and could choose whether to volunteer the reasoning underlying their views. Where discussion resulted in a revised consensus statement, the facilitator invited a further round of anonymous voting to assess agreement with the revised wording, where time permitted within the scheduled session. Consensus was predefined as >80% agreement, consistent with thresholds commonly reported in health census studies [30].

### Consensus Reporting

Following each focus group session, the first author (ST) reviewed the session recording, transcript, anonymous voting results, and facilitator notes to prepare a postsession summary. Each summary documented: (1) the consensus statement, (2) the initial agreement achieved, and (3) key discussion points, particularly where differing perspectives were expressed and the outcome of revoting along with the revised statement if applicable. These summaries were subsequently reviewed by all authors before being consolidated into a single table to compare findings across all focus group sessions.

The consolidated table was used to report levels of agreement to highlight divergent participant perspectives across participant groups. Statements meeting the predefined consensus threshold were distinguished from statements that did not.

Member checking was not undertaken following the focus groups. Participants did not review transcripts or preliminary findings, as the modified vNGT incorporated iterative clarification, discussion, and repeat voting during the focus group sessions, allowing participants to refine and validate consensus statements in real time.

Following completion of the consensus reporting, the findings were interpreted through the lens of SDT. SDT was applied as an interpretive framework to examine how the identified consensus statements related to the psychological needs of autonomy, competence, and relatedness, thereby aiding the interpretation of the findings within the existing behavior change literature. Note that SDT was not used to guide coding, theme development, or the consensus-building process.

## Results

### Overview

Recruitment occurred between February and August 2025. Of the 34 participants who showed interest in participating in the study, 14 participants were excluded due to being unreachable, unresponsive, or declining to participate in the online focus group. Of the 21 participants who completed the survey, 20 participants progressed to the 5 online focus groups (via Zoom) conducted between August 2025 and September 2025. Participants reported experience across multiple stakeholder groups, with health care professionals representing the largest group (16/21, 76%). Government or nonprofit experience was reported by 33% (7/21), pharmaceutical industry experience by 19% (4/21), technology industry experience by 24% (5/21), and digital health academia by 14% (3/21). Self-reported affiliations were not mutually exclusive. The majority of participants also reported having more than 5 years of experience in the digital health sector (15/21, 71%). Details on participant characteristics were collected and tabulated (Table 1).

**Table 1:** Characteristics of the participants (N=21).

Characteristics			Value, n (%)
**Self-reported experience and affiliations^a^**			
	Medical and health care professionals	16 (76)	
	Tech industry	5 (24)	
	Pharmaceutical industry	4 (19)	
	Other private health-relevant industries	1 (5)	
	Government and nonprofit organizations	7 (33)	
	Academics in digital health	3 (14)	
**Years of experience in the digital health sector**			
	1-2	0 (0)	
	2-4	6 (29)	
	5-10	12 (57)	
	>10	3 (14)	

^a^Self-reported experience and affiliations were not mutually exclusive. Participants could select multiple current and previous professional roles.

### Phase 1: Survey Results

#### Quantitative Questions: Participant Ratings

Across the 5 prompts, agreement was strongest for aligning gamification and financial incentives with patients’ individual needs (prompt 1), with all participants selecting “agree” or “strongly agree.” Participants also generally supported allowing users to customize gamification and incentive features (prompt 5). In contrast, perspectives on financial incentives (prompt 2), whether gamification increased app complexity and reduced reliability (prompt 3), and concerns regarding data sharing (prompt 4) were more divided, with responses showing greater variability.

All prompts had median ratings of 5 or higher, indicating neutral to generally positive agreement overall. Overall, these findings suggest broad agreement on the importance of personalization, alongside greater uncertainty regarding the potential risks and trade-offs associated with gamification and financial incentives. Figure 1 illustrates the categorized Likert Scale ratings of patient perspective prompts as evaluated by the participants.

**Figure 1:**
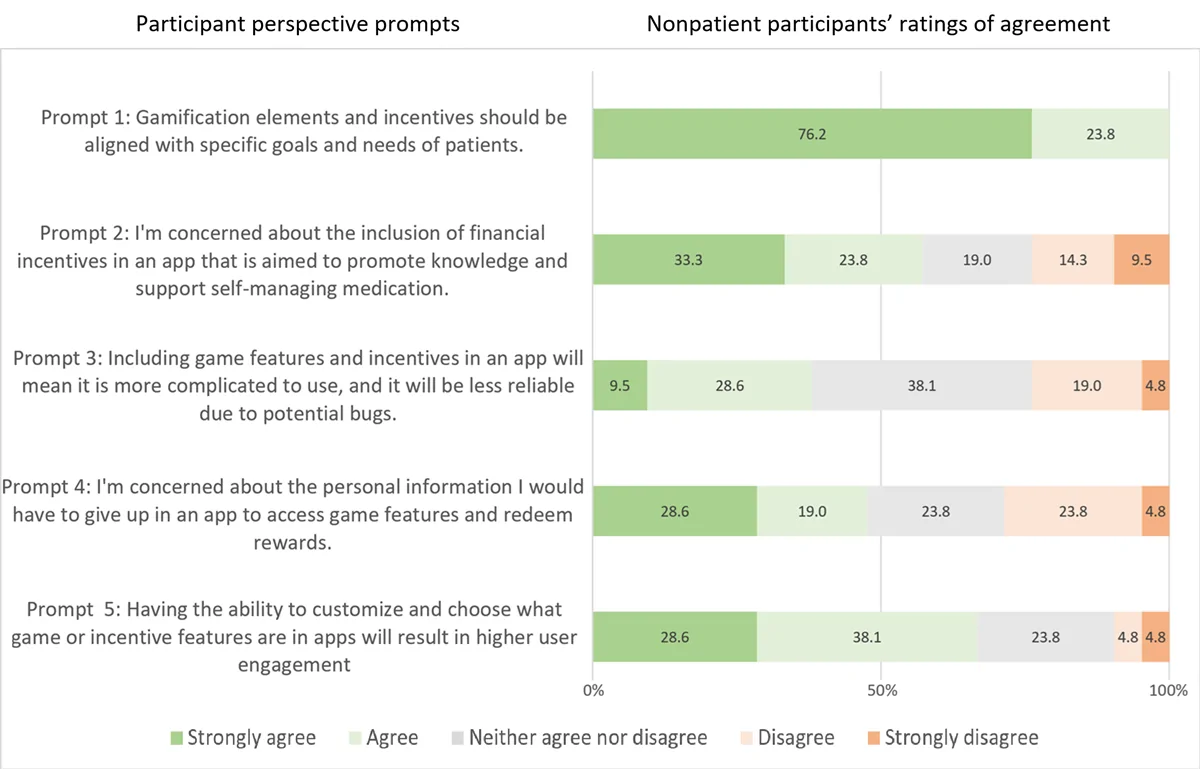
Likert Scale of patient perspective prompts rated by participants.

#### Qualitative Questions: Thematic Analysis

##### Overview

All 21 participants provided responses to each of the 11 open-ended survey questions. Participants provided responses to each question ranging from brief comments to detailed reflections, with many responses comprising multiple sentences or paragraphs. There were 6 themes arising from the analysis. These themes, descriptions of themes, and representative quotations are listed below.

##### Tailored Gamification for Adherence

Gamification was widely viewed as a promising strategy to improve medication adherence, particularly when tailored to individual preferences and clinical contexts. Participants highlighted features such as progress tracking, avatars, and symbolic feedback, while emphasizing that gamification should remain simple and avoid trivializing chronic health conditions:

Point-based systems, rewards and progress tracking. I think these elements would be engaging and trigger a person’s reward sensors.

Participant 3

##### Financial Incentives: Contested Motivator

Financial incentives were met with mixed reactions. While some participants saw them as effective for initial engagement, others raised concerns about ethical implications, sustainability, and unintended consequences such as addiction or overcompliance. The perceived value and structure of incentives were seen as critical to their success:

Any financial incentive will increase engagement. Yet, that does not mean you achieve your goal of retaining medication adherence. It will not encourage a behavioral change but rather a reward system where it is expected to receive a financial reward for “good” behavior. Once the incentive is removed, the effect will fade away.

Participant 13

##### Designing for Diversity and Inclusion

Participants emphasized that mHealth interventions should accommodate diverse patient populations, considering differences in age, digital literacy, health literacy, and clinical needs. Co-design and user testing were viewed as important strategies for improving inclusiveness:

Addressing usability would require co-design with patient cohorts, and then user testing throughout the process.

Participant 4

##### Usability Barriers to Engagement

Barriers such as poor digital literacy, time burden, and complexity were frequently cited. Participants stressed the need for simple, intuitive interfaces and low setup barriers to support sustained engagement, especially among older adults and those with chronic conditions:

Gamification may be more difficult for less tech-savvy populations who may find the format frustrating to use.

Participant 12

##### Trust Through Data Governance

Trust in data handling emerged as a central concern. Participants called for transparent privacy policies, opt-in consent, and adherence to national and international standards. These expectations were considered applicable to all app features, including financial incentives, with participants not identifying any additional governance requirements specific to gamified or incentive-based interventions:

Data security, privacy and governance should follow healthcare industry standards. Consider ISO 27001 data security principles.

Participant 4

##### Validated and Sustainable Innovation

Participants emphasized the need for evidence-based development, including pilot studies, benchmarking, and minimum viable product testing. Sustainability, both technical and financial, was seen as essential for long-term success and user retention:

Governance, healthcare professional sign-off, patient advocate groups, clinical studies proving effectiveness in a controlled environment, real-world evidence, etc.

Participant 9

A table with additional quotes for each theme is available in Multimedia Appendix 6.

### Phase 2: Focus Group Results

#### Overview

A total of 24 consensus statements were developed from the 6 themes identified in Phase 1 (Table 2).

**Table 2:** Consensus statements organized by theme.

Theme	Consensus statement
Theme 1: Tailored gamification for adherence	Gamification is most effective when aligned with users’ motivational drivers. Gamification in adherence apps may unintentionally trivialize chronic health conditions. Design must balance engagement with respect for the seriousness of users’ health experiences. Symbolic progress and personalization foster emotional investment and routine formation. Modular design and simplicity ensure accessibility and relevance.
Theme 2: Financial incentives (contested motivator)	Financial incentives can be effective for initial engagement. The impact of financial incentives may not be sustained once the incentives are withdrawn. Ethical implications and sustainability are critical considerations for financial incentives. Financial incentives may lead to unintended consequences such as addiction or over-compliance. There is a critical need for design frameworks that prevent unintended consequences from financial incentives. The value and structure (e.g. amount, frequency & method of redemption) of financial incentives are crucial to their success. Non-monetary rewards, such as virtual tokens or points, can be used to foster intrinsic motivation and engagement without the risks associated with financial incentives.
Theme 3: Designing for diversity and inclusion	Co-design with patient cohorts is recommended to ensure relevance and inclusivity. Inclusive design requires early and ongoing engagement with target users. Tailoring app features to diverse user groups are essential for usability and engagement.
Theme 4: Usability barriers to engagement	Poor digital literacy is a significant barrier to engagement. Ease of use is critical to adoption. Time burden and complexity can deter users from sustained engagement. Adherence apps must minimize cognitive & technical load.
Theme 5: Trust through data governance	Gamified and incentivized adherence apps must adhere to the same standards of data privacy, security, and regulatory compliance as all other mHealtha apps. Clear communication and accessible information about data collection, usage, and storage are essential to mitigate user concerns and foster informed participation.
Theme 6: Validated and sustainable innovation	Gamified and incentivized adherence apps, like other mHealth apps, must be grounded in real-world evidence to ensure clinical effectiveness. Scalability should be built into the design to support broader implementation across diverse patient populations and settings. Ongoing user feedback and regular updates are essential to sustaining long-term adherence outcomes.

^a^mHealth: mobile health.

Initial agreement exceeded the predefined consensus threshold (>80%) for 21 of the 24 statements, with 13 statements achieving unanimous agreement (100%) prior to discussion.

Overall, participants demonstrated broad agreement that if used, gamification and financial incentives should be implemented using a patient-centered, evidence-based approach. Consensus supported personalizing gamification to individual users through customizable and modular designs, while ensuring accessibility through co-design and consideration of varying levels of digital and health literacy. Participants also agreed on the importance of transparent data governance, privacy protections, and ongoing evaluation to support the safe and sustainable implementation of mHealth interventions. Although financial incentives were recognized as having potential value, participants consistently emphasized that their design should minimize unintended consequences and be supported by appropriate ethical safeguards. Multimedia Appendix 7 provides the consensus statements’ initial agreement rates and discussion summaries.

Three statements (statements 2, 12, and 19) did not initially achieve the predefined consensus rate and generated substantial discussion. These discussions provided additional insight into areas where participants identified important design considerations and trade-offs.

#### Statement 2: Gamification in Adherence Apps May Unintentionally Trivialize Chronic Health Conditions

A minority of participants across 4 focus groups (5/20, 25%) initially disagreed with the statement, arguing that gamification and trivialization were unrelated. The discussions revealed that gamification has the potential to overemphasize adherence behaviors at the expense of the condition itself. This may lead some users to perceive their condition as less serious (than possibly desirable) when it is represented through game-like elements. A mismatch between an app’s representation of a condition and lived experience could create a dissonance that may have unintended consequences. Some participants highlighted generational differences, noting that older populations were more likely to perceive gamification as trivial, whereas younger audiences are generally more accustomed to such features.

Across all focus groups, unanimous agreement was ultimately reached in support of the statement. Participants highlighted that this potential risk exists and should be carefully considered and mitigated during app design.

#### Statement 12: Nonmonetary Rewards, Such as Virtual Tokens or Points, Can Be Used to Foster Intrinsic Motivation and Engagement Without the Risks Associated With Financial Incentives

Some participants across 3 focus groups (4/20, 20%) expressed skepticism regarding the long-term effectiveness of virtual tokens and points. While there was general agreement that nonmonetary rewards can support intrinsic motivation and user engagement, discussions highlighted that these mechanisms are not without limitations. Participants noted that motivation may diminish once rewards are exhausted, raising concerns about sustainability over time. Additionally, some participants cautioned that nonmonetary rewards could become problematic if they acquire perceived tangible value, potentially introducing addictive dynamics.

The groups concluded that although nonmonetary rewards present fewer risks than financial incentives, their design and implementation require careful consideration. Participants emphasized the importance of structuring these systems to sustain engagement while minimizing unintended consequences, even if the overall risk profile is comparatively lower.

#### Statement 19: Adherence Apps Must Minimize Cognitive and Technical Load

Four participants across 3 focus groups (4/20, 20%) challenged the notion of fully minimizing cognitive load, emphasizing that a certain degree of cognitive engagement is necessary to ensure meaningful interaction with the app and the reliability of self-reported data. Discussions further highlighted that excessive simplification may undermine user engagement and reduce the perceived value of the app. Participants in one of the focus groups advocated for maintaining cognitive engagement, noting its role in supporting health literacy and fostering user autonomy. However, they also stressed the need to balance information provision carefully to avoid overwhelming users.

The discussions of the 3 focus groups indicate that while reducing cognitive and technical demands may improve usability, an optimal balance must be maintained. Participants underscored the importance of designing flexible systems that accommodate varying user needs, promoting both accessibility and meaningful engagement without compromising health literacy.

## Discussion

### Summary of Results and Principal Findings

This study provides insight into how a predominantly health care professional sample of nonpatient stakeholders perceive the use of gamification and financial incentives in mHealth apps for medication adherence and the considerations perceived to influence their implementation. Overall, participants emphasized the importance of patient-centered design, particularly personalization, simplicity, privacy, and transparent use of incentives, while also highlighting broader considerations relating to ethics, sustainability, and implementation within health care systems.

Participants also expressed differing views regarding the role of financial incentives, the potential impact of gamification on app complexity, and the balance between personalization and scalability. Although opinions initially varied, discussions identified shared priorities around user involvement in design, ethical governance, simplicity, and evidence-informed implementation, which informed the subsequent interpretation of the findings.

### Comparison of Patient and Nonpatient Stakeholder Perspectives

Across both phases of the study, the predominantly health care professional sample of nonpatient stakeholders demonstrated substantial alignment with previously reported patient perspectives, particularly regarding the importance of personalization, simplicity, privacy, and the careful use of incentives in medication adherence apps [11]. This convergence is consistent with the broader mHealth literature, where patients across diverse digital health settings consistently identify personalization, usability, and trust as key determinants of engagement and acceptability [11-15,31]. This suggests that these principles represent fundamental design considerations for patient-centered digital health interventions rather than features unique to medication adherence apps. The observed alignment likely reflects the increasing emphasis on patient-centered design within digital health, while many nonpatient stakeholders may also draw on their own lived experiences as patients [32,33].

Despite this overall alignment, nonpatient stakeholder perspectives revealed greater nuance in their consideration of technical complexity, data sharing, and the role of financial incentives. While patients primarily emphasized usability, low burden, and trust in relation to their own experiences using digital health technologies [11,14,15], stakeholders more frequently considered how these priorities could be operationalized within health care systems. This was reflected in greater emphasis on governance, scalability, integration with existing clinical workflows, and the generation of real-world evidence to support implementation [34,35]. Similarly, although both stakeholder groups recognized the potential value of financial incentives, nonpatient stakeholders placed greater emphasis on their ethical implementation, long-term sustainability, and potential unintended consequences, while viewing concerns such as increased complexity or trivialization as challenges of poor design rather than inherent limitations of gamification [36]. While individual nonpatient participants occasionally drew on their professional expertise during discussions, their contributions generally reflected differences in emphasis rather than fundamentally different perspectives. This convergence suggests that the identified design principles resonate across multiple stakeholder groups involved in the design, implementation, and evaluation of medication adherence interventions, supporting their potential applicability within multidisciplinary digital health settings.

Overall, these findings suggest that patient and stakeholder perspectives are complementary rather than competing. Patient perspectives help define the characteristics required for engagement and acceptability, whereas stakeholder perspectives extend these priorities by considering feasibility, governance, and sustainable implementation within health care systems. Integrating both perspectives may therefore support the development of gamified and incentivized mHealth interventions that are not only acceptable to end users but are also feasible to implement, evaluate, and sustain in routine practice.

### Interpreting the Findings Through SDT

Much like the patient perspectives identified in our earlier study [11], the findings align closely with the core principles of SDT, providing a useful lens for interpreting nonpatient stakeholder perspectives on gamification and financial incentives in medication adherence apps [10]. Across both the initial reflections and the consensus statements, nonpatient stakeholders consistently emphasized design features that support autonomy through personalization, user choice, transparency, and co-design, as well as competence, reflected in calls for simplicity, reduced cognitive load, clear indicators of progress, and evidence-based functionality. Relatedness was less prominent but nonetheless evident in the value placed on human touchpoints, inclusivity, and integration with existing health care systems. These findings are consistent with MedVenture (University of Colorado), an SDT-based gamified medication adherence app that operationalizes autonomy, competence, and relatedness through features such as personalized medication planning, tailored feedback and encouragement, and social support mechanisms [37].

Consistent with SDT [10], financial incentives were viewed as potentially effective for initiating engagement but insufficient and at times counterproductive for sustaining long-term behavior change, particularly when perceived as controlling or poorly designed. Rather than rejecting incentives outright, participants highlighted the importance of using them cautiously and ethically, as short-term enablers within broader autonomy-supportive environments [8,10]. Together, these findings suggest that gamified adherence interventions are most likely to support the internalization of medication-taking behaviors when they prioritize autonomy and competence, incorporate opportunities for relational support, and align motivational strategies with both user needs and system-level requirements such as privacy, regulation, and scalability.

### Key Analytical Contributions and Insights

This study offers several analytical contributions that extend beyond existing patient-centered and effectiveness-focused discussions of gamification and financial incentives in mHealth interventions. First, the views of the expert stakeholders challenge the assumption that reducing cognitive effort is always beneficial for engagement and adherence [11]. While participants consistently emphasized the importance of simplicity and low user burden, many also highlighted that some degree of cognitive engagement is necessary to support health literacy, meaningful interaction, and the internalization of medication-taking behaviors. This tension suggests that overly simplified designs may risk undermining users’ sense of competence and informed agency, indicating a need for more subtle design approaches that balance ease of use with sufficient challenge to promote learning and sustained engagement [38]. This is central to Bandura’s theory of self-efficacy and consistent with principles in video game design, in which challenge is deliberately adjusted to the user’s ability to provide an optimal experience and facilitate motivation [39-41].

A second novel insight concerns the perceived risk of trivializing chronic health conditions through gamification. Here, trivialization refers to reducing complex, burdensome health experiences into overly simplistic, entertainment-oriented interactions that may downplay their seriousness, emotional impact, or lived realities. Rather than viewing trivialization as an inherent consequence of gamification, nonpatient stakeholders framed it as a failure of design quality and contextual sensitivity. Participants emphasized that when gamification is superficial or poorly aligned with users’ lived experiences, it may undermine perceptions of illness severity; however, when thoughtfully designed, gamification can support engagement without compromising respect for users’ health contexts. This reframing shifts the focus from whether gamification should be used in adherence interventions to how it should be implemented responsibly, reinforcing calls within the broader gamification literature for theory-informed, context-aware, and ethically grounded design [34].

Finally, the study contributes a less explored perspective on the risks associated with financial incentives. Beyond common concerns about the decay of engagement once incentives are withdrawn or the undermining of intrinsic motivation [10,42], participants highlighted the potential for incentives to drive excessive or maladaptive behaviors, including overcompliance and compulsive engagement. These risks were understood to be shaped by the magnitude, frequency, and framing of rewards, drawing attention to parallels with reward-driven digital systems more broadly [43]. By foregrounding the possibility that incentives can be harmful not only when they are ineffective but also when they are overly effective, the findings underscore the importance of robust design frameworks that explicitly address ethical boundaries, safeguard against unintended consequences, and support sustainable, autonomy-aligned engagement strategies in gamified adherence apps [44].

### Implications for mHealth Design, Policy, and Practice

The findings have clinically informed implications for the design, governance, and implementation of gamified and incentivized mHealth interventions for medication adherence. From a design perspective, stakeholders’ emphasis on personalization, simplicity, and ethical use of incentives highlights the need for flexible, modular app architectures that can accommodate diverse user needs without increasing cognitive or technical burden [45,46]. Developers could prioritize autonomy-supportive features, such as optional gamification elements, user-controlled settings, and transparent data practices, while ensuring that interfaces remain intuitive and accessible to users with varying levels of digital literacy [10,47]. Additionally, simplicity should not be equated with minimal engagement; instead, designs should balance ease of use with sufficient cognitive engagement to support health literacy, competence, and meaningful interaction over time [39-41,48].

From a policy and governance standpoint, the strong consensus around privacy, security, and ethical responsibility underscores that gamified adherence apps must be held to the same regulatory and clinical standards as other mHealth interventions [49]. Financial incentives, in particular, require clear ethical frameworks to guide decisions about reward value, frequency, and duration, and to mitigate risks such as dependency, overcompliance, or inequitable access [36,50]. These findings suggest that policymakers and commissioning bodies may wish to consider establishing guidance that supports the responsible use of incentives as short-term engagement tools rather than long-term behavioral drivers, while encouraging transparency around data use and accountability for unintended consequences [51]. Embedding evaluation requirements, including the collection of real-world evidence, may further support trust and legitimacy in these interventions [52].

In practice, the findings suggest that successful implementation of gamified and incentivized adherence apps depends on early and sustained involvement of target user groups and integration within existing health care systems [53,54]. Co-design with patient cohorts can help ensure relevance, inclusivity, and acceptability, while ongoing feedback mechanisms enable iterative refinement as user needs evolve [54]. Integration with clinical workflows and health care providers may also enhance continuity of care and reinforce adherence behaviors beyond the app itself [55]. These implications point to the need for mHealth interventions that are not only engaging and innovative but also ethically grounded, evidence-informed, and embedded within broader health system infrastructures to support sustainable medication adherence [46,53].

However, given the predominance of health care professionals within the participant sample, these implications should be interpreted as predominantly clinically informed perspectives rather than a comprehensive representation of the wider stakeholder ecosystem. Further work involving policymakers, payers, technology developers, and other commercial stakeholders is needed to establish broader consensus regarding policy, reimbursement, and implementation priorities.

### Limitations

This study has 3 key limitations that should be considered when interpreting the findings. First, although purposive sampling sought to recruit participants with diverse expertise across the digital health ecosystem, the sample was predominantly comprised of participants with health care professional experience. While many participants held multidisciplinary roles spanning clinical practice, research, digital health, and implementation, the perspectives elicited may nevertheless reflect a stronger clinical orientation than those of other stakeholder groups, such as policymakers, payers, technology developers, behavioral economists, or digital ethicists. Although participants represented diverse stakeholder backgrounds, the study was designed to develop consensus across the collective sample rather than compare perspectives between stakeholder groups. As such, differences within or between stakeholder groups were not examined systematically. Furthermore, participants were recruited within the Australian health care context. Consequently, while the patient-centered design principles identified are broadly consistent with the international mHealth literature and may therefore have broader relevance, findings relating to governance, reimbursement, regulation, and health system integration may be most transferable to health care systems with similar organizational and policy contexts. Although this expertise strengthened discussion around clinical feasibility, ethics, and implementation, future research should incorporate greater representation from underrepresented stakeholder groups to establish whether similar priorities emerge across the wider digital health ecosystem.

Second, the study relied on a virtual format and a modified nominal group technique, which, although well suited to structured consensus-building, may have constrained spontaneous interaction and limited the exploration of more nuanced or contested views. The iterative consensus-building process, including discussion informed by aggregated voting results between rounds, may also have encouraged convergence of opinions through conformity or social desirability bias, whereby participants moderated or aligned their views in response to the emerging group perspective. Where revised consensus statements were proposed late in the scheduled session, practical time constraints may have limited opportunities for additional rounds of voting, introducing minor procedural variation across the focus group sessions. Consequently, the final consensus statements should be interpreted as reflecting negotiated agreement among participants rather than entirely independent judgments, and minority or dissenting perspectives may have been underrepresented despite efforts to capture individual views prior to group discussion. Additionally, participation was limited to stakeholders who were willing and able to engage in online sessions, potentially introducing self-selection bias.

Finally, the use of AI in the thematic analysis introduces potential limitations. Model outputs are dependent on prompt design and may reflect latent biases present in training data. There is also a risk of oversimplification or loss of contextual awareness when generating themes [56]. To mitigate these risks, AI outputs were used solely to support preliminary coding and theme generation and were systematically reviewed, refined, and confirmed against the original dataset.

### Directions for Future Research

Future research should examine how varying levels of cognitive engagement influence health literacy, motivation, and adherence outcomes in gamified mHealth interventions, with particular attention to balancing simplicity and meaningful challenge across users with differing digital literacy and health needs. Longitudinal and experimental studies are also needed to better understand the short- and long-term effects of financial incentives, including underexplored risks such as overcompliance, compulsive engagement, and dependency, as well as differences between monetary and nonmonetary rewards and their design characteristics. In parallel, implementation-focused research should explore how gamified adherence apps can be effectively integrated within health care systems, regulatory frameworks, and reimbursement models, using co-design approaches and real-world evidence to inform scalable, sustainable, and ethically grounded deployment.

### Conclusion

This study provides novel insight into the perspectives of a predominantly health care professional–experienced sample of nonpatient stakeholders on the use of gamification and financial incentives in mHealth apps for medication adherence, complementing and extending previously reported patient views. The findings demonstrate strong alignment between patients and nonpatient stakeholders on core priorities, including personalization, simplicity, privacy, and the ethical use of incentives, while also revealing important differences in emphasis related to feasibility, sustainability, and system-level considerations. Through a structured consensus-building process, initially diverse perspectives converged on shared principles that emphasize user-centered, transparent, and ethically grounded design.

Interpreted through the lens of SDT, the findings highlight the importance of supporting autonomy, competence, and relatedness to promote the internalization of adherence behaviors and sustain engagement over time. At the same time, the study identifies several novel contributions, including the need to balance simplicity with meaningful cognitive engagement, the framing of trivialization as a design failure rather than an inherent limitation of gamification, and the recognition that financial incentives may pose risks of overengagement as well as motivation decay. Overall, these insights underscore that the successful use of gamification and incentives in mHealth adherence interventions depends not on whether such strategies are used, but on how they are designed, governed, and embedded within broader health care systems.

## Appendix

Multimedia Appendix 1 High level study overview.

Multimedia Appendix 2 COREQ checklist.

Multimedia Appendix 3 Full survey.

Multimedia Appendix 4 AI prompts.

Multimedia Appendix 5 Facilitator guide.

Multimedia Appendix 6 Additional quotes by themes.

Multimedia Appendix 7 Focus group results.

## Abbreviations

COREQ: Consolidated Criteria for Reporting Qualitative Research
HREC: Human Research Ethics Committee
mHealth: mobile health
SDT: self-determination theory
vNGT: virtual nominal group technique

## Data Availability

The datasets comprising participant responses generated and analyzed during this study are not publicly available due to ethical and confidentiality considerations. Public archiving of the data was not considered appropriate because of participant confidentiality, the potential impact on participants’ willingness to provide authentic responses, and intellectual property and commercial-in-confidence considerations. Consistent with the approved ethics protocol and participant consent, the data are not available for public release or sharing with third parties. Accordingly, requests for access to the data cannot be accommodated.

## Generative AI Use

The authors acknowledge the use of OpenAI’s GPT-5 via Microsoft Copilot in the development of this manuscript. Copilot was used to assist in the thematic analysis process, including the verification of coding structures and the identification of preliminary themes based on human-coded data. It was also used to support the drafting and refinement of the facilitator guide. Additionally, Copilot contributed to enhancing the clarity of language and restructuring of sentences throughout the manuscript. All content generated with AI assistance was critically reviewed and revised by the authors, who assumed full responsibility for the accuracy and integrity of the final manuscript.
