# Supplementary material for: Nonpatient Stakeholder Perspectives on the Use of Gamification and Financial Incentives in Mobile Health for Medication Adherence: Modified Nominal Group Technique Study
Source: J Med Internet Res. 2026 Oct 29;28:e101295. doi: 10.2196/101295 (PMC13760330; doi:10.2196/101295)
Supplement: Supplementary file 2 [file jmir_v28i1e101295_app2.pdf]

Multimedia Appendix 2: COREQ (COnsolidated criteria for REporting Qualitative research) Checklist applied to "Non-Patient Stakeholder Perspectives on the use of Gamification and Financial Incentives in mHealth for Medication Adherence: Mixed Methods Consensus Study"

Topic	Item No.	Guide Questions/Description	Reported in section / Remarks
**Domain 1: Research team and reflexivity**			
Personal characteristics			
Interviewer/facilitator	1	Which author/s conducted the interview or focus group?	Methods: Phase 2: Focus Groups Sessions (*Online focus group sessions, each comprising four to six participants, were conducted and facilitated by the first author (ST).*)
Credentials	2	What were the researcher's credentials? E.g. PhD, MD	Declaration section/Author contributions (*ST (male; BPharm), a pharmaceutical industry professional with qualitative research experience, conceptualized and designed the study, facilitated participant recruitment and data collection, conducted initial coding and thematic analysis, and led the drafting of the manuscript. SC (male; BPharm, MSc, PhD, Grad Cert Edu [Higher Ed], AACP, FPS, FHEA), an associate professor, contributed to study design, participated in coding calibration, reviewed thematic analysis, and critically revised the manuscript. LS (female; BA [Hons] [Psych], PhD), a professor, contributed to study design, participated in coding calibration, reviewed thematic analysis, and provided critical revisions to the manuscript.)*
Occupation	3	What was their occupation at the time of the study?	
Gender	4	Was the researcher male or female?	
Experience and training	5	What experience or training did the researcher have?	
Relationship with participants			
Relationship established	6	Was a relationship established prior to study commencement?	Declaration section/Author contributions (*Some participants had prior or ongoing professional relationships with one or more of the researchers before the commencement of the study. These relationships were general in nature and*
Participant knowledge of the interviewer	7	What did the participants know about the researcher? e.g. personal goals, reasons for doing the research	

			unrelated to the study's design, conduct, or outcomes. No participants had personal relationships with the researchers.)
Interviewer characteristics	8	What characteristics were reported about the inter viewer/facilitator? e.g. Bias, assumptions, reasons and interests in the research topic	The participants were aware that the facilitator had personal motivations for conducting the study including that the research would contribute to a higher degree.
**Domain 2: Study design**			
Theoretical framework			
Methodological orientation and Theory	9	What methodological orientation was stated to underpin the study? e.g. grounded theory, discourse analysis, ethnography, phenomenology, content analysis	Methods: A modified Nominal Group Technique with thematic analysis.
Participant selection			
Sampling	10	How were participants selected? e.g. purposive, convenience, consecutive, snowball	Combination of purposive & convenience sampling strategies utilized i.e. via Direct Outreach via Public and Professional Networks (via email, phone or LinkedIn) and Social media and Professional Platforms (via LinkedIn).
Method of approach	11	How were participants approached? e.g. face-to-face, telephone, mail, email	
Sample size	12	How many participants were in the study?	Results (*Of the 34 participants who showed interest in participating in the study, 14 participants were excluded due to being unreachable or unresponsive or declining to participate in the online focus group. Of the 21 participants who completed the survey, 20 participants progressed to the 5 online focus groups (via Zoom) conducted between August 2025 and September 2025.*)
Non-participation	13	How many people refused to participate or dropped out? Reasons?	
Setting			
Setting of data collection	14	Where was the data collected? e.g. home, clinic, workplace	Methods Phase 1: Pre-session Survey. Data was collected in the online secure REDCap survey link.<br><br>Methods Phase 2: Online Focus Group Sessions (*The sessions were recorded and transcribed using Zoom.*)

Presence of non-participants	15	Was anyone else present besides the participants and researchers?	Apart from the first author and participants, there were no other parties in any of the focus groups.
Description of sample	16	What are the important characteristics of the sample? e.g. demographic<br>data, date	Results (*Of the 21 participants who completed the survey, 20 participants progressed to the 5 online focus groups (via Zoom) conducted between August 2025 and September 2025. Participants reported experience across multiple stakeholder groups, with healthcare professionals representing the largest group (16/21, 76%). Government or non-profit experience was reported by 33% (7/21), pharmaceutical industry experience by 19% (4/21), technology industry experience by 24% (5/21), and digital health academia by 14% (3/21). Self-reported affiliations were not mutually exclusive. The majority of participants also reported to have more than 5 years of experience in the digital health sector (15/21, 71%). Details on participant characteristics (Table 1) were collected and tabulated.)*
Data collection			
Interview guide	17	Were questions, prompts, guides provided by the authors? Was it pilot tested?	Methods: *The instrument was developed specifically for this study and was informed by prior research on patient perspectives on the same topic [11]. Survey items (Quantitative and Qualitative questions) elicited demographic/background as well as attitudes toward gamification and financial incentives in mHealth apps for medication adherence. For the demographic section, participants were able to select multiple stakeholder categories to reflect their current and past professional experiences, as well as their years of experience. Participants' perspectives were explored using quantitative rating scales and open-ended questions. Patient-derived perspectives were incorporated as prompts to enable comparison between*

			stakeholder and prior patient viewpoints. These prompts were designed to capture key domains identified in prior research, including personalization of gamification and incentives, motivational impact, usability and accessibility, ethical considerations, and data governance. The survey was not piloted, pre-tested with participants, nor was it formally validated. However, all authors reviewed the survey prior to administration to establish content and face validity. The survey is provided in Multimedia Appendix 3.<br><br>*The focus groups followed a facilitator guide incorporating a standardized discussion protocol, in which each proposed consensus statement was presented sequentially for discussion and voting. The facilitator guide is provided in Multimedia Appendix 5.*
Repeat interviews	18	Were repeat interviews carried out? If yes, how many?	All participants who carried out the survey also participated in the focus group sessions except for 1 participant who dropped out for the focus group session.
Audio/visual recording	19	Did the research use audio or visual recording to collect the data?	Methods Phase 2: Focus Group Sessions (*The sessions were recorded and transcribed using Zoom.*) Visual and audio recording were used to collect the data.
Field notes	20	Were field notes made during and/or after the interview or focus group?	Notes were made by the first author after the focus groups sessions to annotate comments for consideration during the analysis.
Duration	21	What was the duration of the interviews or focus group?	All focus groups were limited to a duration of one hour.
Data saturation	22	Was data saturation discussed?	Methods: Ideal consolidation (*During the analytical process, themes were compared to the raw data iteratively to identify the emergence of new concepts relevant to the study objectives. Recruitment ceased when the three most recent survey responses did not contribute any additional*

			concepts beyond those already identified, suggesting diminishing conceptual novelty and that sufficient breadth of perspectives had been captured to inform the subsequent focus group discussions. [29].)
Transcripts returned	23	Were transcripts returned to participants for comment and/or correction?	The transcripts were not returned to the participants however the summary results of the survey were discussed in the focus group sessions, and they were invited for the post study analysis summary of results.
**Domain 3: analysis and findings**			
Data analysis			
Number of data coders	24	How many data coders coded the data?	Methods: Idea consolidation (*All authors (ST, SC, LS) initially conducted familiarization exercises on the survey responses. A subset of responses was independently coded by each author to calibrate coding strategies. Coding differences were discussed collaboratively to refine the coding framework and achieve agreement among the authors. The remaining data were coded by ST using NVivo (Release 1.7.2), with subsequent review by SC and LS to ensure consistency of coding. Codes were then organized into preliminary thematic nodes using Microsoft Excel (Version 2506). Coding decisions, theme development, and subsequent refinements were documented throughout the analysis to provide an audit trail.*)
Description of the coding tree	25	Did authors provide a description of the coding tree?	Inductive coding approach used to develop the coding tree.<br>Methods: Idea consolidation (*All authors (ST, SC, LS) initially conducted familiarization exercises on the survey responses. A subset of responses was independently coded by each author to calibrate coding strategies. Coding differences were discussed collaboratively to refine the coding*

			framework and achieve agreement among the authors. The remaining data were coded by ST using NVivo (Release 1.7.2), with subsequent review by SC and LS to ensure consistency of coding. Codes were then organized into preliminary thematic nodes using Microsoft Excel (Version 2506).)
Derivation of themes	26	Were themes identified in advance or derived from the data?	Themes derived from the data.<br>Methods: Idea consolidation (*AI then conducted theme identification (Step 3), which was cross validated against the authors' manual node groupings. Any differences in interpretation were resolved through discussion and review of the original data. No substantive differences in theme structure or interpretation were identified. A second round of theme identification confirmed the same six themes.*)
Software	27	What software, if applicable, was used to manage the data?	REDCap – Survey data collection<br>Zoom – Audio and visual recording and transcription<br>NVivo (Release 1.7.2) – Transcription to codes<br>Microsoft Excel (Version 2506) – Codes to nodes/themes<br>OpenAI's GPT-5 (Microsoft Copilot) – Support thematic analysis with deidentified data.
Participant checking	28	Did participants provide feedback on the findings?	The transcripts were not returned to the participants however the summary results of the survey were discussed in the focus group sessions, and they were invited for the post study analysis summary of results.
Reporting			
Quotations presented	29	Were participant quotations presented to illustrate the themes/findings?<br>Was each quotation identified? e.g. participant number	Yes, Multimedia Appendix 6 provides additional illustrative quotes for each theme with each quotation identified with the participant number.
Data and findings consistent	30	Was there consistency between the data presented and the findings?	Yes, there is consistency between the data and findings outlined in the Results & Discussion section.

Clarity of major themes	31	Were major themes clearly presented in the findings?	Yes, Multimedia Appendix 6 outlines the major themes identified in the thematic analysis.
Clarity of minor themes	32	Is there a description of diverse cases or discussion of minor themes?	Yes, the results text under “*Qualitative questions: Thematic analysis”* provides a descriptive explanation for each theme to support consensus statements in multimedia appendix.

Reference: Tong A, Sainsbury P, Craig J. Consolidated criteria for reporting qualitative research (COREQ): a 32-item checklist for interviews and focus groups. *International Journal for Quality in Health Care*. 2007. Volume 19, Number 6: pp. 349 – 357
