# Supplementary material for: Nonpatient Stakeholder Perspectives on the Use of Gamification and Financial Incentives in Mobile Health for Medication Adherence: Modified Nominal Group Technique Study
Source: J Med Internet Res. 2026 Oct 29;28:e101295. doi: 10.2196/101295 (PMC13760330; doi:10.2196/101295)
Supplement: Supplementary file 3 [file jmir_v28i1e101295_app3.pdf]

# Bridging Perspectives: Stakeholder Engagement for Mobile Health Applications Utilising Gamification and Incentives to Improve Medication Adherence

---

Researchers at the University of Sydney School of pharmacy are looking for individuals aged 18 and above located in Australia with a background, career experiences or affiliations within digital health domain to partake in our study.

The study is comprised of two components; an exploratory pre-session panel questionnaire and a group panel discussion that may be facilitated online or in person. The total amount of time should not exceed 1.5 hours. The data collected during the group will be analysed to identify potential opportunities, barriers, and limitations to the use of mobile apps utilising gamification and incentives to address medication adherence. The results will be publicly disseminated.

Examples of relevant professions include medical/healthcare professionals, health related societies/guilds/associations, private companies, government/non-profit organizations, academics and health ethicists/lawyers.

To confirm your eligibility and register your interest in participating, please complete this online screening form. The screening form will enable you to also book a convenient time to speak with a member of the research team to discuss the study. This online screening form should take no more than 5 minutes followed by a pre-session panel questionnaire that should not take more than 20 minutes.

For more details about this study please read the Participant Information Statement before deciding whether to participate.

[Attachment: "Participant_Information_Statement.docx"]

---

1) ☐ I confirm that I have reviewed the Participant Information Statement.

## Participant Screening

| | | |
|---|---|---|
| 2) | I am aged 18 years or older | ◯ Yes<br>◯ No |
| 3) | I am located in Australia | ◯ Yes<br>◯ No |
| 4) | I am an individual with a background, career experience or affiliation within the health/medical and/or tech domain. | ◯ Yes<br>◯ No |

# Bridging Perspectives: Stakeholder Engagement for Mobile Health Applications Utilising Gamification and Incentives to Improve Medication Adherence

## Consent

I agree to take part in this research study. In giving my consent, I confirm that that:
• The details of my involvement have been explained to me, and I have been provided with a written Participant Information Statement to keep.
• I understand the purpose of the study is to investigate perspectives on the use of mobile apps utilising gamification and incentives to improve medication adherence.
• I acknowledge that the risks and benefits of participating in this study have been explained to me to my satisfaction.
• I understand that in this study I will be required to participate in an online focus group.
• I understand that my participation will be audio recorded.
• I understand that my participation will be video recorded.
• I understand that all recordings will only be used for the purpose of this study and will be deleted after analysis.
• I understand that being in this study is completely voluntary.
• I am assured that my decision to participate will not have any impact on my relationship with the research team or the University of Sydney.
• I understand that I am free to withdraw from this study at any time and that it will not be possible to withdraw my individual comments from the focus group records.
• I have been informed that the confidentiality of the information I provide will be protected and will only be used for purposes that I have agreed to. I understand that information about me will only be told to others with my permission, except as required by law.
• I understand that the results of this study may be published, and that publications will not contain my name or any identifiable information about me.

| | |
|---|---|
| I consent to recordings (audio) | ◯ Yes<br>◯ No |
| I consent to recordings (video) | ◯ Yes<br>◯ No |
| I consent to being contacted for future studies | ◯ Yes<br>◯ No |
| I would like feedback on the overall results of this study | ◯ Yes<br>◯ No |
| Full Name | ______________________ |
| Phone Number | ______________________ |
| Email Address | ______________________ |

| | |
|---|---|
| Which day(s) of the week would be best to contact you via phone? | ☐ Any<br>☐ Monday<br>☐ Tuesday<br>☐ Wednesday<br>☐ Thursday<br>☐ Friday<br>☐ Saturday<br>☐ Sunday |
| Which day(s) of the week would you prefer the focus group to be on? | ☐ Any<br>☐ Monday<br>☐ Tuesday<br>☐ Wednesday<br>☐ Thursday<br>☐ Friday<br>☐ Saturday<br>☐ Sunday |
| What time of day would you be available? | ☐ Morning (9am - 12pm)<br>☐ Noon (12 - 3pm)<br>☐ Evening (3 - 6pm)<br>☐ Other |
| Please specify preferred time: | __________________________________ |

# Bridging Perspectives: Stakeholder Engagement for Mobile Health Applications Utilising Gamification and Incentives to Improve Medication Adherence

## Background questions and pre-session panel questionnaire

Data collected in this questionnaire is de-identified and aggregated for research analysis.
All responses cannot be traced back to individuals.

1) Relevant past or current experience/affiliations within Health or Tech domain

- ☐ Medical/Healthcare Professionals
- ☐ Medical societies/guilds/associations
- ☐ Tech industry
- ☐ Pharmaceutical industry
- ☐ Other private health relevant industries
- ☐ Government/non-profit organizations
- ☐ Academics
- ☐ Health/digital lawyers and ethicist
- ☐ Other

2) Years of experience in either health or tech domain?

- ◯ 1-2
- ◯ 2-4
- ◯ 5-10
- ◯ more than 10

Gamification and financial incentives in mobile apps offer promising avenues to bolster medication adherence among patients, addressing poor adherence rates prevalent even in high-income countries. By integrating game-like elements such as challenges, point-based systems, rewards, and progress tracking, these apps can transform the multi-faceted challenge of medication adherence into an engaging and motivating experience for patients. Furthermore, financial incentives provide tangible rewards for adherence, incentivizing patients to stick to their medication regimens consistently. This approach not only can foster better medication adherence but facilitates the option for healthcare providers to remotely monitor and intervene, when necessary, thereby improving overall health outcomes. For pharmaceutical companies, such initiatives can enhance brand loyalty and market share by positioning their products within innovative and patient-centric digital solutions. Most importantly, patients themselves stand to benefit from increased adherence, leading to better health outcomes and potentially reduced healthcare costs in the long term.

[Attachment: "Tran S et al. (2022) The Use of Gamification and Incentives in Mobile Health Apps to Improve Medication Adherence.pdf"]

3) What are your thoughts about integrating gamification into mobile apps for medication adherence?

__________________________________

4) Which game elements do you think could work well for encouraging medication adherence? Please also include your reasoning behind your response.

__________________________________

5) From your viewpoint, how effective do you think financial incentives could be for encouraging medication adherence in mobile apps? Please also include your reasoning behind your response.

__________________________________

6) What specific concerns, if any, do you harbor regarding the utilization of financial incentives in this context of promoting medication adherence through mobile apps?

__________________________________

7) What factors would influence your decision to include gamification, financial incentives, or a mix of both to improve medication adherence through mobile apps? __________________________________________

Our most recent study, delving into patient perspectives on the use of gamification and incentives into mobile apps aimed at addressing medication adherence, identified three themes. Firstly, "purpose-driven design" emerged as a cornerstone, emphasizing the necessity for app developers to align gamification elements and incentive structures with the specific goals and needs of patients. Participants expressed that apps that allow users access to game features, which are designed to promote knowledge about medicines, and allow the self-monitoring of medication taking were broadly accepted and even desirable

Secondly, the importance of "trust-based standards" became evident, underlining the significance of transparent and secure systems to foster patient trust in the app's functionalities and data handling practices particularly in the distribution of financial incentives. The notion that a user's behaviour would be monitored by a third party and that users may accrue financial or nonfinancial benefits as rewards for adherent behaviour created some scepticism. Lastly, "personal choice" emerged as a crucial aspect, advocating for app customization options that empower individuals to tailor their adherence journey according to their preferences and lifestyle to game features and financial incentive offerings. Participants believed that the adoption of apps with game features and incentives will require that users maintain a high level of personal choice in the selection of app features, desired engagement levels, and ways to redeem rewards.

These themes collectively underscore the nuanced considerations essential for the effective implementation of gamification and incentives to support medication adherence in mobile health apps from a patients' perspective.

It is essential to consider perspectives from industry experts, regulatory bodies, and community stakeholders to ensure a well-rounded approach when considering patient perspectives. Based on your professional experience and the information above, we aim to prioritize these themes and gather best practice insights.

[Attachment: "Tran S et al.  (2024) Understanding Patient Perspectives on the Use of Gamification and Incentives in mHealth Apps to Improve Medication Adherence Qualitative Study.pdf"]

8) Gamification elements and incentives should be aligned with specific goals and needs of patients.

Strongly Disagree — Strongly Agree

*(Place a mark on the scale above)*

9) Would it be feasible to identify what game features are relevant to a general cohort of patients for goal setting or empowerment and if so, how would you go about it? __________________________________________

10) I'm concerned about the inclusion of financial incentives in an app that is aimed to promote knowledge and support self-managing medication.

Strongly Disagree — Strongly Agree

*(Place a mark on the scale above)*

11) What concerns do you have and how would you incorporate incentives and rewards in a way that addresses your concerns? __________________________________________

12) Including game features and incentives in an app will mean it is more complicated to use and it will be less reliable due to potential bugs.

Strongly Disagree — Strongly Agree

*(Place a mark on the scale above)*

13) What development and testing strategies would you use when incorporating game or incentive features into a medication management app to support reliability and usability?

__________________________________

14) I'm concerned about the personal information I would have to give up in an app to access game features and redeem rewards.

Strongly Disagree — Strongly Agree

*(Place a mark on the scale above)*

15) What standards and best practices should be employed for data security and governance?

__________________________________

16) How can security standards and best practices be transparently shown to app users to instil a sense of security?

__________________________________

17) Having the ability to customize and choose what game or incentive features are in apps will result in higher user engagement

Strongly Disagree — Strongly Agree

*(Place a mark on the scale above)*

18) Is an app with static features or modular components more likely to succeed and why?

__________________________________
