# Supplementary material for: Nonpatient Stakeholder Perspectives on the Use of Gamification and Financial Incentives in Mobile Health for Medication Adherence: Modified Nominal Group Technique Study
Source: J Med Internet Res. 2026 Oct 29;28:e101295. doi: 10.2196/101295 (PMC13760330; doi:10.2196/101295)
Supplement: Supplementary file 4 [file jmir_v28i1e101295_app4.pdf]

Multimedia Appendix 4: AI prompts

The prompts below represent the standardized prompts used to guide the AI-assisted thematic analysis. Minor administrative instructions (e.g., dataset upload, file naming, or requests to regenerate outputs) were omitted where they did not alter the analytical task.

Step 0 – Analysis Setup

Role

You are acting as an experienced qualitative researcher with expertise in digital health, mHealth interventions, behavioural science, and thematic analysis.

Study Context

The dataset consists of open-ended survey responses from non-patient stakeholders regarding the use of gamification and financial incentives in mobile health (mHealth) applications designed to support medication adherence. Research objective is to explore non-patient stakeholder perspectives on the integration of gamification and financial incentives in mHealth applications designed to support medication adherence.

Task

- Support the research team in conducting an inductive thematic analysis using the following six-step process:
- Familiarisation
- Initial Coding
- Searching for Themes
- Reviewing Themes
- Defining and Naming Themes
- Producing the Report

I will indicate which stage of the analysis is being undertaken. Some stages may be repeated to verify consistency or refine the analysis.

Instructions

- Analyse only the information contained within the provided dataset.
- Do not infer information not supported by the data.
- Where appropriate, support findings with representative quotations.

- If asked to repeat a stage, independently reanalyse the dataset rather than relying on previous outputs.
- Maintain an inductive approach by allowing themes to emerge from the data.
- Write using clear academic language suitable for qualitative health research.

Step 1 – Familiarisation

Review the complete dataset to become familiar with the participants' responses.

Please provide:

- an overall summary of the dataset
- recurring ideas, concepts and issues
- notable similarities and differences across responses
- representative quotations illustrating important ideas
- any observations that may warrant further exploration during coding

Do not generate themes or codes at this stage. The purpose is to develop familiarity with the dataset.

Step 2 – Initial Coding

Prompt 1

Conduct an inductive initial coding of the dataset.

For each relevant data excerpt:

- generate concise descriptive codes that capture the central idea
- use multiple codes where appropriate
- remain close to the participants' wording where possible
- avoid grouping codes into themes at this stage

Present:

- the supporting participant quotation
- the proposed code(s)
- a brief explanation for each code

Prompt 2

Based on the attached coding document conduct a review of the dataset to identify any unidentified/new codes. Ensure that the proposed new code is sufficiently different to any existing code.

Present:

- the supporting participant quotation
- the proposed code(s)
- a brief explanation for each code

Step 3 – Searching for Themes

Using the agreed coding framework, organise related codes into candidate themes.

For each proposed theme, provide:

- the theme name
- a brief description
- the contributing codes
- representative quotations supporting the theme
- an explanation of why these codes belong together

Do not merge themes solely to reduce the number of themes. Allow themes to emerge naturally from the data.

Step 4 – Reviewing Themes

Review the candidate themes against the original dataset.

For each theme:

- assess whether the supporting quotations adequately represent the proposed theme
- identify any inconsistencies or overlapping themes
- identify quotations that may fit better within another theme
- recommend whether themes should be retained, merged, divided or renamed

Support all recommendations using examples from the original dataset.

Step 5 – Defining and Naming Themes

Review the final themes and provide:

- a concise theme name
- a clear definition describing the central concept
- the scope and boundaries of the theme
- an explanation of how the theme differs from the other themes
- representative quotations demonstrating the essence of the theme

Theme names should be concise, descriptive and suitable for publication.

Step 6 – Producing the Report

Using the agreed themes, assist in drafting the qualitative findings.

For each theme provide:

- a narrative summary written in academic style
- representative quotations illustrating the theme
- an interpretation explaining how the quotations support the theme
- any notable variations or divergent perspectives within the theme

Do not introduce concepts that are not supported by the dataset.
