# Supplementary material for: Nonpatient Stakeholder Perspectives on the Use of Gamification and Financial Incentives in Mobile Health for Medication Adherence: Modified Nominal Group Technique Study
Source: J Med Internet Res. 2026 Oct 29;28:e101295. doi: 10.2196/101295 (PMC13760330; doi:10.2196/101295)
Supplement: Supplementary file 5 [file jmir_v28i1e101295_app5.pdf]

Multimedia Appendix 5: Focus group facilitator guide

Outline

- **Introduce moderator and assistant (if present) & topic/purpose of the focus group (t=0)**

  Good morning/afternoon/evening and welcome to research session. Thank you for taking the time to join us to talk about use of mobile apps containing game features or rewards to address medication adherence. My name is Steven Tran and assisting me is __________. We're both/I am with the University of Sydney conducting this research as part of my PhD. You were invited here today because you have been identified as individuals with relevant experience to the research topic. We would like discuss responses provided in the pre-session survey and gain consensus. The results will provide valuable insight into improving patient health outcomes by addressing medication adherence through mobile applications utilizing gamification and financial incentives, and support in the formulation of a best practice document.

- **Outline guidelines for session (t=1)**
  - No right or wrong answers
  - Listen respectfully to others as they share their views, we may ask if you agree or disagree and why afterwards
  - The session is being recorded, one person speaking at a time
  - For Zoom calls: We would prefer you to show your camera however it we respect your decision should you choose not to use it. Video will be used to help with analysis of quotes (i.e. confirm quotes) and will be deleted after the completion of the study.
  - Preferred name basis always

    - Should you need to briefly leave the focus group e.g. phone call, please do so quietly and rejoin as quickly as possible.

START RECORDING NOW

- Present the themes identified from the pre-session survey and invite initial comments.
- Introduction to each consensus statement under each theme
    - Participants anonymously vote (agree/disagree) on each proposed consensus statement. (t=15)
- Discussion protocol
    - Present each consensus statement and share the anonymous voting results.
        - If unanimous agreement was achieved: Invite additional comments or clarifications.
        - If unanimous agreement was achieved:
            - invite participants to explain the reasons for disagreement.
            - Encourage discussion among participants. Use probing prompts where clarification or elaboration was required (e.g., "Could you tell me more about what has informed your perspective?").
            - Revote following discussion where statements were revised or clarification was required.
- *Thank you very much for your time and the information you shared today.* (t=55)
    - Ask if participants would be interested in getting feedback in the form of a summary after the study is complete. (Record the names of interested participants for future dissemination of the study summary, irrespective of previous response on REDCap)
