# Supplementary material for: Nonpatient Stakeholder Perspectives on the Use of Gamification and Financial Incentives in Mobile Health for Medication Adherence: Modified Nominal Group Technique Study
Source: J Med Internet Res. 2026 Oct 29;28:e101295. doi: 10.2196/101295 (PMC13760330; doi:10.2196/101295)
Supplement: Supplementary file 6 [file jmir_v28i1e101295_app6.pdf]

Multimedia Appendix 6: Additional quotes by themes

Theme 1: Tailored Gamification for Adherence

- *"Point-based systems, rewards and progress tracking. I think these elements would be engaging and trigger a person's reward sensors."* [Participant 3]
- *"Gamification can hold a valuable role in engaging patients and ensuring medication adherence however I am weary of them being designed and marketed by pharmaceutical companies rather than patient driven as this could also lead to the counter effect of over compliance and an inability for step-down or deprescribing of treatment where it would be more appropriate to step down."* [Participant 6]
- *"I think a system similar to the 'closing of the rings' would be useful. Every day you'll start with something that's incomplete. You'll have to take all your medicine to complete the ring or whichever design is chosen."* [Participant 5]
- *"Modular components so you can advance and adapt with different trends."* [Participant 3]
- *"Having an online pet or a plant that gets fed or grows if you take the medication and log it into an app."* [Participant 1]

Theme 2: Financial Incentives – Contested Motivator

- *"Financial incentives can be very effective for encouraging medication adherence in mobile apps as most people enjoy financial reward for time. However, the financial incentive must be directly aligned with/justify the time taken to use the app."* [Participant 12]
- *"Financial incentives would allow people to come back and engage. Without financial incentives there would be a lack of continuity in the gamified experience." [Participant 2]*
- *"Monetary incentives carry risks such as creating a dependency on external rewards rather than fostering intrinsic motivation, potential inequities among patient populations, and the likelihood that adherence may drop once the incentives are withdrawn."* [Participant 6]
- *"As a dual registered pharmacist and GP who has seen the effects and cost of addictions including gambling, adding a monetary incentive is a dangerous strategy that may cause an array of other mental health and addiction issues."* [Participant 6]
- *"There would need to be a way of policing the incentives to ensure people don't take advantage of it."* [Participant 19]
- *"Depends on the value - would have to be moderate for the effort involved to adhere."* [Participant 7]
- *"There are ethical, fun, and easier ways to give rewards here e.g., a 'MediCoin' that you accrue by reading medication facts, or by completing streaks, or by logging into the app for 30 days in a row."* [Participant 1]

Theme 3: Designing for Diversity and Inclusion

- *"It would need to be piloted and studied with evidence indicating positive outcomes and regard reviews to ensure applicability."* [Participant 6]
- *"Yes, a short survey provided for specific groups/populations would help identify which game features would be useful. I think elderly patients would also be less inclined if they are technologically challenged."* [Participant 10]
- *"It is important to be inclusive when designing the app. It should be for different literacy levels, languages, and cognitive abilities to ensure broad usability."* [Participant 18]

Theme 4: Usability Barriers to Engagement

- *"It is a very interesting concept and I think it would ensure consumers pay more attention to their medications. It might also improve compliance but quite often elderly people take medications and they are not always well-versed in using a smart phone so it may actually be a limiting step."* [Participant 19]
- *"An easy-to-navigate app. UX should be a priority. An app that you can start in seconds and be fully into the game/experience is important."* [Participant 14]

- *"Time burden and complexity can deter users from sustained engagement."* [Participant 18]
- *"Adherence apps must minimize cognitive & technical load."* [Participant 18]

Theme 5: Trust Through Data Governance

- *"If you are taking healthcare information, should use HIPAA / Australian standards e.g. ensure the servers are on Australian soil, ensure the data is encrypted at rest, ensure accounts are password protected etc."* [Participant 1]
- *"Any app which holds personal healthcare data must follow the usual principles around storage of data particularly sensitive health information. Considerations around the potential for cybersecurity threats need to be taken into account giving this is a rising area of concern."* [Participant 8]
- *"Provide links to all data privacy, security and governance principles, explicitly explaining what personal data is being collected, who it is being shared with, if it is being sold on etc."* [Participant 4]
- *"Display certifications like ISO 27001 and compliance with Australian Privacy Principles on your app's website and within the app. Provide a clear privacy policy detailing data handling and security measures. Regularly publish security update logs and third-party audit results to demonstrate ongoing commitment to user data protection."* [Participant 13]

Theme 6: Validated and Sustainable Innovation

- *"Do a pilot study or research of patient for a condition where compliance is poorest and see if gaming features make a difference."* [Participant 6]
- *"Modular components with regular game updates and new features would be more effective for patients that need medications over a longer period. I wonder what would happen if they no longer needed medications but wanted to continue playing the game."* [Participant 20]
- *"Use agile development for iterative testing, conduct user acceptance testing with target users, and implement continuous monitoring to quickly identify and fix issues."* [Participant 13]
- *"Good Q&A testers. Nimble coding team to iterate on the app and patch bugs quickly."* [Participant 14]
