# Supplementary material for: Nonpatient Stakeholder Perspectives on the Use of Gamification and Financial Incentives in Mobile Health for Medication Adherence: Modified Nominal Group Technique Study
Source: J Med Internet Res. 2026 Oct 29;28:e101295. doi: 10.2196/101295 (PMC13760330; doi:10.2196/101295)
Supplement: Supplementary file 7 [file jmir_v28i1e101295_app7.pdf]

Multimedia Appendix 7: Focus Group agreement rates and discussion summaries

Theme 1: Tailored Gamification for Adherence		
	**Initial agreement rates**	**Discussion following initially consensus polling**
**Consensus statement 1: Gamification is most effective when aligned with users' motivational drivers.**	18/20 (90%)	A participant in Focus group #2 felt that the term "most effective" was conditional as some users might have poor or toxic motivational drivers, such as addiction issues. The group eventually recommended that the statement was modified to "Gamification is most effective when aligned with motivational drivers that are beneficial to users' health outcomes.". This modified statement was unanimously accepted by the group.<br><br>Another participant in Focus group #5 suggested that motivational drivers alone might not ensure app usage. They argued that gamification itself could serve as a driver, and alignment with users' existing motivations might not be as critical as ensuring gamification leads to positive health outcomes, such as medication adherence. They also noted the complexity of identifying and quantifying motivational drivers, which can evolve over time. Whilst unanimous consensus was not reached, the group recommended balancing gamification with positive motivational drivers and health contexts.<br><br>Overall, participants believe that while aligning gamification with users' motivational drivers is important, it is crucial to consider the nature of these drivers and the broader context in which gamification is applied. Modifying the statement to reflect these considerations could make it more agreeable and applicable across different user groups.
**Consensus statement 2: Gamification in adherence apps may unintentionally trivialize chronic health conditions.**	15/20 (75%)	Some participants in Focus groups #2 and #3 initially disagreed, feeling that gamification and trivialization were unrelated. However, they acknowledged that linking a health condition to a game could downplay its seriousness, focusing more on adherence than the condition itself. One of the participants in group #3 also noted that it is specifically poor design and application of gamification that could result in trivializing chronic health conditions. Unanimous consensus was reached in both groups.<br><br>Similarly, Focus group #4 and #5 also reached unanimous consensus after participants highlighted generational differences. Older populations potentially find gamified mHealth apps surprising or trivializing, while younger generations tend to be more accustomed to such technology. Both groups agreed that while gamification encourages adherence, it might oversimplify complex chronic conditions.<br><br>Overall, the discussions suggest that while gamification can be effective in promoting adherence, careful consideration of design and target audience is essential to avoid trivializing serious health conditions.

**Consensus statement 3: Design must balance engagement with respect for the seriousness of users' health experiences.**	19/20 (95%)	In Focus group #5, participants discussed the subjective nature of "seriousness," noting that perceptions vary widely between patients and healthcare professionals. For example, a patient with diabetes might view their condition as primarily a matter of managing sugar intake, while healthcare professionals recognize the potential for serious complications if poorly managed. The group agreed that while it is important for app design to remain respectful of these experiences, the primary goal is to increase medication adherence, which requires high engagement. They debated whether balancing engagement with respect for seriousness is always necessary, suggesting that prioritizing effectiveness may be more important in some cases. Some participants also felt that attempting to accommodate all interpretations of seriousness could limit the app's overall effectiveness.<br><br>Overall, the discussions indicate that while balancing engagement with respect for the seriousness of health experiences is desirable, it may not always be practical. Participants emphasized that the primary focus should remain on developing an engaging and effective app that achieves its intended outcomes.
**Consensus statement 4: Symbolic progress and personalization foster emotional investment and routine formation.**	18/20 (90%)	Some participants in both Focus group #1 and #3 argued that while symbolic progress and personalization are important, they are not sufficient on their own. They emphasized the need for additional elements such as engagement, involvement, and nudges to effectively form habits and routines. They also highlighted the importance of incorporating a human aspect, rather than relying solely on automated responses from a chatbot.<br><br>Overall, the discussions suggest that while symbolic progress and personalization are key factors in fostering emotional investment and routine formation, they should be complemented by other engagement strategies and tailored to individual needs. Providing concrete examples and incorporating a human touch could enhance the effectiveness and applicability of the statement.
**Consensus statement 5: Modular design and simplicity ensure accessibility and relevance.**	20/20 (100%)	This statement highlights the importance of designing mHealth apps in a way that is both modular and simple, ensuring that they are accessible to a wide range of users and remain relevant to their needs. This design philosophy not only makes the app easier to navigate and use but also allows for customization and adaptability to meet diverse user requirements.

Theme 2: Financial Incentives – Contested Motivator		
	**Initial agreement rates**	**Discussion following initially consensus polling**
**Consensus statement 6: Financial incentives can be effective for initial engagement.**	20/20 (100%)	Offering financial incentives can successfully attract users and encourage them to engage with an app or program initially. The unanimous consensus underscores the effectiveness of this strategy in capturing users' attention and motivating them to take the first step towards engagement. This approach can be particularly useful in overcoming initial barriers to participation and fostering a positive start to the user experience.
**Consensus statement 7: The impact of financial incentives may not be sustained once the incentives are withdrawn.**	20/20 (100%)	While financial incentives can be highly effective in driving initial engagement, their influence tends to diminish once the financial incentives are no longer available. This highlights the importance of considering long-term engagement strategies beyond financial incentives to ensure sustained user participation and adherence. This insight is crucial for designing interventions that maintain their effectiveness over time, even after the initial financial motivators are removed.
**Consensus statement 8: Ethical implications and sustainability are critical considerations for financial incentives.**	20/20 (100%)	It is crucial to ensure that their use is ethically sound and sustainable over time. This consideration is vital for maintaining trust, fairness, and the overall integrity of health programs, ensuring that they benefit users without causing unintended negative consequences.
**Consensus statement 9: Financial incentives may lead to unintended consequences such as addiction or over-compliance.**	17/20 (85%)	A participant in Focus groups #1, #4, and #5 had similar opposing opinions. They indicated that the unintended consequences could be mitigated through careful consideration on the design and implementation of financial incentives. An example of this is that excessive incentives, such as valuable items or frequent rewards, could lead to negative outcomes. The discussions suggest that specifying the conditions under which these risks might occur could make the statement more agreeable and applicable.
**Consensus statement 10: There is a critical need for design frameworks that prevent unintended consequences from financial incentives.**	20/20 (100%)	This unanimous consensus statement highlights the importance of ensuring that financial incentives do not lead to unintended consequences such as addiction, over-compliance, or other adverse outcomes. It underscores the necessity for careful planning, ethical considerations, and sustainable practices in the design of financial incentive programs to maximize their benefits while minimizing potential risks.

**Consensus statement 11: The value and structure (e.g. amount, frequency & method of redemption) of financial incentives are crucial to their success.**	19/20 (95%)	In Focus group #5, a participant argued the importance of the specifics of financial incentives, noting that while they are significant, they might not be the sole determinants of success. The participant further highlighted that users might initially engage due to the incentives but may continue using the app because they find it enjoyable or beneficial. The group eventually reached consensus that the value and structure of financial incentives play a significant role in the success of the incentive feature itself.
**Consensus statement 12: Non-monetary rewards, such as virtual tokens or points, can be used to foster intrinsic motivation and engagement without the risks associated with financial incentives.**	16/20 (80%)	Some participants in Focus groups #1, #2, and #5 discussed the effectiveness of non-monetary rewards. While some were skeptical about the long-term impact of virtual tokens and points, others sought clarification on their redeemability for tangible rewards. They agreed that non-monetary rewards could foster intrinsic motivation and engagement but noted that these rewards carry fewer risks than financial incentives.<br><br>Concerns were raised about the sustainability of motivation once all rewards are obtained and the potential for non-monetary rewards to be addictive if linked to tangible value.<br><br>The groups concluded that while non-monetary rewards can be effective, their design and implementation need careful consideration, similar to financial incentives, to ensure sustained engagement and minimize potential unintended consequences, albeit at a lower risk.

**Theme 3: Designing for Diversity and Inclusion**		
	**Initial agreement rates**	**Discussion following initially consensus polling**
**Consensus statement 13: Co-design with patient cohorts is recommended to ensure relevance and inclusivity.**	20/20 (100%)	Participants unanimously agree that co-designing with patient cohorts can lead to more effective and user-centered solutions. By incorporating the perspectives and experiences of patients, designers can ensure that the interventions address real needs, are culturally sensitive, and are more likely to be accepted and used by the target population. This approach fosters a sense of ownership and empowerment among patients, ultimately contributing to better health outcomes.
**Consensus statement 14: Inclusive design requires early and ongoing engagement with target users.**	20/20 (100%)	Continuous engagement with users helps to identify and address potential issues early on, fosters a sense of ownership, and ensures that the design remains relevant and accessible. This approach not only enhances the usability and effectiveness of the product but also builds trust and satisfaction among the users, ultimately leading to better outcomes.

**Consensus statement 15: Tailoring app features to diverse user groups are essential for usability and engagement.**	18/20 (90%)	In Focus group #5, participants discussed the challenges of designing an app that caters to all user groups. They noted that an app designed for one demographic, such as younger users, would look very different from one designed for older users. They argued that trying to create a one-size-fits-all app would be ineffective. Instead, they suggested focusing on creating a minimum viable product (MVP) that can be tailored to specific groups over time. They acknowledged that while it might not be possible to achieve 100% coverage, aiming for 80% of the population is a more practical and impactful approach. However, they also emphasized the importance of being hyper-focused on specific demographics to ensure the app meets their needs effectively.<br><br>The discussions indicate that while tailoring app features to diverse user groups is essential, it is also important to balance this with practical considerations. Creating an MVP and iteratively tailoring it to specific groups can help achieve a noticeable impact while ensuring the app remains relevant and engaging for its users.

**Theme 4: Usability Barriers to Engagement**		
	**Initial agreement rates**	**Discussion following initially consensus polling**
**Consensus statement 16: Poor digital literacy is a significant barrier to engagement.**	20/20 (100%)	Unanimous consensus was achieved in all focus groups, recognizing that addressing digital literacy is essential for ensuring that all users can effectively utilize and benefit from an mHealth app. This insight emphasizes the need for designing user-friendly interfaces, providing adequate support and education, and considering the varying levels of digital literacy among the target population to enhance overall engagement and accessibility.
**Consensus statement 17: Ease of use is critical to adoption.**	20/20 (100%)	If an app is not easy to use, it is unlikely to be adopted by users, regardless of its potential benefits. This insight emphasizes the need for simplicity in design, clear navigation, and a seamless user experience to ensure that users can easily engage with and benefit from the app. Prioritizing ease of use is essential for maximizing adoption rates and achieving the desired health outcomes.
**Consensus statement 18: Time burden and complexity can deter users from sustained engagement.**	20/20 (100%)	There is a need for designing interventions that are straightforward, easy to use, and respectful of users' time to ensure sustained engagement and adherence. By minimizing time burden and complexity, designers can create more user-friendly and effective health interventions.

**Consensus statement 19: Adherence apps must minimize cognitive & technical load.**	16/20 (80%)	Some participants in Focus Group #2 & #5 raised concerns about completely minimizing cognitive load and emphasized the necessity of some level of cognitive engagement to ensure meaningful interaction with the app and the reliability of the data collected.<br><br>Similarly, Focus Group #4 participants advocated cognitive engagement to improve health literacy and autonomy, while also stressing the importance of balancing information provision to avoid overload. They recommended flexibility in the app's demands to ensure usability and promote health literacy.<br><br>Overall, the discussions suggest that while reducing cognitive and technical demands can enhance usability, it is essential to maintain a balance that promotes genuine engagement and health literacy.

**Theme 5: Trust Through Data Governance**		
	**Initial agreement rates**	**Discussion following initially consensus polling**
**Consensus statement 20: Gamified and incentivized adherence apps must adhere to the same standards of data privacy, security, and regulatory compliance as all other mHealth apps.**	20/20 (100%)	Despite achieving unanimous consensus, several focus groups (Focus groups #1 to #4) provided additional considerations regarding the importance of ensuring that gamified and incentivized adherence applications adhere to the same rigorous standards for data privacy, security, and regulatory compliance as other mHealth apps. Participants concurred that these apps should meet or exceed existing regulatory requirements, with a particular emphasis on protecting personally identifiable information and ensuring it is not shared with advertising companies. Furthermore, participants highlighted app specifications that warrant further qualification & registration as a medical device and the challenges associated with maintaining compliance amidst constantly evolving regulations.
**Consensus statement 21: Clear communication and accessible information about data collection, usage, and storage are essential to mitigate user concerns and foster informed participation.**	20/20 (100%)	Clear, accessible information about data practices is crucial for building trust and encouraging informed participation. By ensuring that users are fully aware of how their data is collected, used, and stored, developers can address potential concerns and foster a sense of security and confidence in the app. This approach not only enhances user engagement but also supports ethical standards and regulatory compliance in data management.

Theme 6: Validated and Sustainable Innovation		
	**Initial agreement rates**	**Discussion following initially consensus polling**
**Consensus statement 22: Gamified and incentivized adherence apps, like other mHealth apps, must be grounded in real-world evidence to ensure clinical effectiveness.**	19/20 (95%)	Focus group #5 discussed that while it is important to ground apps in evidence, it may not always be feasible to have comprehensive real-world evidence from the outset. Where this is not feasible, they recommended developing a MVP and trialling it to gather the necessary evidence over time.<br><br>The discussions suggest that while grounding gamified and incentivized adherence apps in real-world evidence is crucial for ensuring clinical effectiveness, it is also important to recognize the practical challenges and adopt a phased approach to evidence gathering. This approach allows for iterative improvements based on real-world data, ultimately enhancing the app's effectiveness and relevance.
**Consensus statement 23: Scalability should be built into the design to support broader implementation across diverse patient populations and settings.**	19/20 (95%)	Participants in Focus group #1 unanimously agreed on the significance of scalability and adaptability but highlighted a crucial missing point: integration with existing medical record systems and healthcare providers (HCPs). They noted that many apps on users' phones do not communicate with each other, creating a technical burden. Integrating with the current medical ecosystem, possibly through QR codes for opt-in or app store access, was seen as essential for a holistic health app.<br><br>A participant in Focus group #5 disagreed with the statement, arguing that a hypercomplex app designed to cover all user groups should be avoided initially. They recommended focusing on creating a hyperfocused MVP and considering scalability as a future enhancement. They agreed that while scalability is important, it should not be the primary focus during the initial development phase. Instead, it should be considered as part of future planning, allowing for the possibility of adding or scaling features as needed.
**Consensus statement 24: Ongoing user feedback and regular updates are essential to sustaining long-term adherence outcomes.**	20/20 (100%)	Regular feedback and updates help address users' evolving needs, preferences, and potential issues, thereby enhancing the app's effectiveness and user satisfaction. By incorporating ongoing feedback and making regular improvements, developers can ensure that the app remains relevant, user-friendly, and capable of delivering sustained adherence outcomes. This approach fosters a dynamic and responsive relationship between the app and its users, ultimately contributing to better health outcomes.
